# Modelling non-stationary annual maximum flood heights in the lower Limpopo River basin of Mozambique

**DOI:** 10.4102/jamba.v8i1.185

**Published:** 2016-05-12

**Authors:** Daniel Maposa, James J. Cochran, Maseka Lesaoana

**Affiliations:** 1 Department of Statistics and Operations Research, University of Limpopo, South Africa; 2 Department of Information Systems, Statistics and Management Science, University of Alabama, United States

## Abstract

In this article we fit a time-dependent generalised extreme value (GEV) distribution to annual maximum flood heights at three sites: Chokwe, Sicacate and Combomune in the lower Limpopo River basin of Mozambique. A GEV distribution is fitted to six annual maximum time series models at each site, namely: annual daily maximum (AM1), annual 2-day maximum (AM2), annual 5-day maximum (AM5), annual 7-day maximum (AM7), annual 10-day maximum (AM10) and annual 30-day maximum (AM30). Non-stationary time-dependent GEV models with a linear trend in location and scale parameters are considered in this study. The results show lack of sufficient evidence to indicate a linear trend in the location parameter at all three sites. On the other hand, the findings in this study reveal strong evidence of the existence of a linear trend in the scale parameter at Combomune and Sicacate, whilst the scale parameter had no significant linear trend at Chokwe. Further investigation in this study also reveals that the location parameter at Sicacate can be modelled by a nonlinear quadratic trend; however, the complexity of the overall model is not worthwhile in fit over a time-homogeneous model. This study shows the importance of extending the time-homogeneous GEV model to incorporate climate change factors such as trend in the lower Limpopo River basin, particularly in this era of global warming and a changing climate.

## Introduction

There is a general notion that the occurrence of extreme events has changed over these recent years and is anticipated to continue to change in terms of intensity, frequency and complexity of the risks. These recent changes are mainly attributed to global warming and natural modes of interannual and interdecadal variability, such as the El Niño phenomenon (Katz 2010; Katz, Parlange & Naveau 2002; Towler *et al*. 2010). The anticipated climate-induced changes are of major concern as they have the potential to render our estimates biased and/or useless, particularly those estimates based on traditional approaches that do not take climate changes into consideration. These probable climate changes can also cause negative societal impacts and disruptions, for instance, destruction of schools, children dropping out of schools leading to early marriages, particularly for girls, and thus creating a vicious poverty circle in the community (Katz 2010; Mudavanhu 2014). According to Katz (2010), previous studies in extreme value theory have shown that the frequency of all forms of extreme events, whether in the form of a single value or a sequence of annual maxima, is more sensitive to variations in the scale parameter (or, in particular, the standard deviation) than to the location parameter (or mean) of a distribution. Cooley (2009) wrote a commentary on the potential application of statistics of extremes to climate change based on the previous work of Wigley.

The annual maximum series (AMS), also known as block maxima, has long been employed to estimate the distribution of extreme events such as flood flows, precipitation and wind speeds. The time-homogeneous generalised extreme value (GEV) distribution, which uses standard properties of the likelihood function, has traditionally been used in designing flood estimation (Coles 2001; Maposa *et al*. 2014b; Towler *et al*. 2010). The use of a stationary GEV distribution assumes that climate changes and all other variables that may affect the validity of the estimation of design floods remain constant over time. In his wisdom, Gumbel (1941:187), the statistician and pioneer in the application of statistics of extremes to hydrology and other various fields, cautioned that:

In order to apply any theory we have to suppose that the data are homogeneous, i.e., no systematical change of climate and important change in the basin have occurred within the observation period and that no such change will take place in the period for which such extrapolations are made. (Katz 2010:71; Katz *et al*. 2002:188)

Without loss of generality, it is easy to understand that the assumption of homogeneity of climatic conditions and other important changes in the basin cannot hold forever. In other words, it is inevitable that climatic conditions change over time. The traditional fitting of the time-homogeneous GEV distribution also assumes that the observations are independent and identically distributed (i.i.d.). According to Katz *et al*. (2002), stationarity implies identicalness and not necessarily independence.

Dr Walter J. Ammann, chairman of the recent International Disaster and Risk Conference held in Davos, Switzerland, 24–28 August 2014, attested that the scope, intensity and complexity of risks as well as the frequency of natural hazards such as floods, earthquakes and forest fires are on the rise in these recent years (IDRC Davos 2014). The increase in the frequency of floods is also supported by a unique survey of 139 national meteorological and hydrological services carried out by the World Meteorological Organisation in 2013, which revealed that floods were the most frequently experienced extreme events worldwide over the course of the decade 2001–2010 (Mudavanhu 2014; WMO 2013). Floods and droughts account for 90% of all the people who are affected by natural disasters (Mudavanhu 2014; Smakhtin 2014). According to Munich Re (2013), the natural catastrophic statistics for the year 2013 was dominated by floods that caused billions of American dollars in losses. In Arya, Boen and Ishiyama (2014), the Director-General of UNESCO, Irina Bokova, stated that:

Every year, more than 200 million people are affected by natural hazards, and the risks are increasing – especially in developing countries, where a single major disaster can set back healthy economic growth for years. As a result, approximately one trillion dollars have been lost in the last decade alone. This is why disaster risk reduction is so essential. Mitigating disasters requires training, capacity building at all levels, and it calls for a change of thinking to shift from post-disaster reaction to pre-disaster action – this is UNESCO’s position. (p. 6)

**FIGURE 1:**
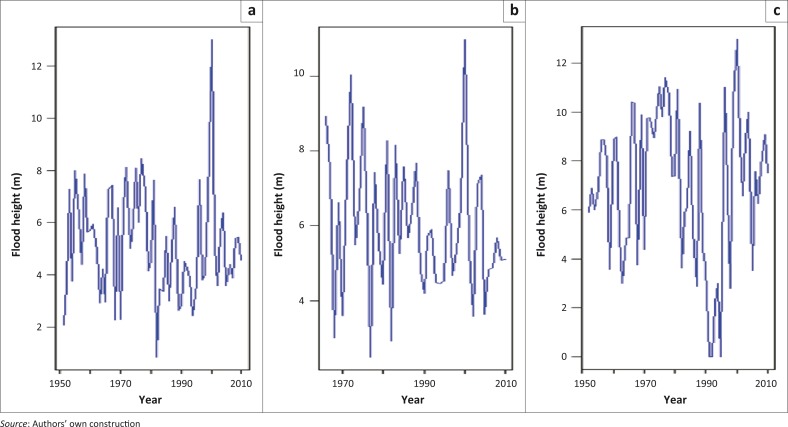
Time series plots of annual daily maximum (AM1) flood heights (in metres) at the three sites: (a) Chokwe (1951–2010), (b) Combomune (1966–2010) and (c) Sicacate (1952–2010) along lower Limpopo River of Mozambique.

Although it is clear from literature that the intensity and frequency of floods have increased over the recent years, it is not clear whether the magnitudes of floods, that is, flood heights, have also increased. If the magnitudes of flood heights have increased over the years, it is expected that the location parameter of the GEV, which is associated with the mean estimate of the distribution, should increase with increase in time. On the other hand, if there is no gradual increase in flood heights over the years and the sporadic extremely high floods are nearly or purely random, then we expect the scale parameter, which is associated with dispersion from the central location, to vary with time (Figure 1). Based on literature cited in the study, there is overwhelming evidence of covariates such as long-term trends or cycles in recent years attributed to man-made activities, which may lead to global warming and atmosphere–ocean circulation patterns such as the El Niño phenomenon, which undermine the long-held traditional assumption of stationarity (Towler *et al*. 2010; Vasiliades, Galiatsatou & Loukas 2015). Evidence of non-stationarity is exhibited in Figure 1 at all the three sites: Chokwe, Combomune and Sicacate of the lower Limpopo River of Mozambique. However, a visual inspection of Figure 1 shows no apparent trend. Figure 1 reveals that in the year 2000 flood height was a very rare extreme event at all the three sites.

The present study considers a non-stationary time-dependent GEV distribution model whose location and scale parameters are expected to vary linearly or nonlinearly with time (Figure 1), whilst the shape parameter remains constant over time. This investigation is carried out for the lower Limpopo River basin (LLRB) of Mozambique. Detailed studies on the goodness of fit of the time-homogeneous GEV distribution in the basin are found in the studies by Maposa, Cochran and Lesaoana (2014a) and Maposa *et al*. (2014b). In the present study, we advocate for a statistical modelling approach based on maximum likelihood (ML) estimation in the possible presence of covariates. These covariates can be in the form of trends, cycles and physical variables such as El Niño (Katz *et al*. 2002). The covariate of particular interest in the present study is the trend. To the best of our knowledge, no similar work in the previous studies relating to statistics of extremes in a changing climate have been done for the LLRB of Mozambique.

According to Katz *et al*. (2002), although the probability weighted moments, also known as L-moments, are more popular in the application of hydrological extremes compared to ML estimation mainly because of their computational simplicity and better performance for small samples where ML is often inconsistent, the probability weighted moment technique has the demerit of being unable to readily incorporate covariates (see also Ferreira & De Haan 2015). On the other hand, the application of ML technique in the presence of covariates is straightforward in both block maxima and peaks-over-threshold (POT) approaches (Katz *et al*. 2002).

The outline of the rest of the article is such that Section 2 presents the research methodology, Section 3 presents the results and discussion of the findings, and finally Section 4 gives the concluding remarks.

## Research methodology

The section presents the sequential steps taken to sort the data into the block maxima series (Ferreira & De Haan 2015), briefly discuss the probability framework of block maxima including the extension of time-homogeneous GEV model to linear and quadratic trend models.

### Study sites and data

Mozambique National Directorate of Water, the authority responsible for water management in Mozambique in the Ministry of Public Works and Housing, provided the data used in the study. The data are hydrometric daily flood heights (in metres) recorded at the sites Chokwe (1951–2010), Combomune (1966–2010) and Sicacate (1952–2010), which are hydrometric stations for the lower Limpopo River of Mozambique (Maposa *et al*. 2014a, 2014b). The three sites are such that Combomune is located in the upper part of the basin about 162 km from the border with South Africa and Zimbabwe, Chokwe is located in the middle of the basin about 130 km downstream of Combomune and Sicacate is further downstream of Chokwe in the lower part of the basin on way to the sea.

### Moving sums and block maxima

The raw data at the three sites were originally recorded as daily flood heights (or water levels). The data records at some sites stretch back to as far as 1930s. However, because of missing values, the records used in the study are for the period 1951–2010 for Chokwe, 1966–2010 for Combomune and 1952–2010 for Sicacate (Figure 1). In order to obtain AMS, sequential steps were taken to obtain the highest flood peak in each hydrological year (or block). Further steps were taken to obtain annual maximum (AM) flood heights of the moving sums of 2, 5, 7, 10 and 30 days. Finally, the following AM time series models were obtained: annual daily maximum (AM1), annual 2-day maximum (AM2), annual 5-day maximum (AM5), annual 7-day maximum (AM7), annual 10-day maximum (AM10) and annual 30-day maximum (AM30). The procedure to obtain these cumulative AM time series models was necessitated by the need to investigate whether the cumulative annual floods have any significant effect on the long-term linear or quadratic trend in location, scale or both.

In statistics of extremes, there are two fundamental approaches used in flood frequency analysis, namely, block maxima (or AMS) and POT (or partial duration series) (Ferreira & De Haan 2015). The approach used in the study is block maxima. In hydrological studies, when sample sizes are large, it is natural to block observations by years (Ferreira & De Haan 2015; Maposa *et al*., 2014b). The data used in this study have sufficiently large AM records extending over 40 years at each site. In flood frequency analysis, the block maxima approach is commonly used ahead of POT when data records have sufficiently large sample sizes and the data quality is adequate (Ferreira & De Haan 2015). The GEV distribution arises naturally when modelling block maxima, whereas for POT the generalised Pareto distribution is commonly used (Ferreira & De Haan 2015).

### Extreme value models

Comprehensive details of probability framework of block maxima and the practical reasons for using block maxima over POT are given by Ferreira and De Haan (2015). Dombry (2013) proved the consistency of ML estimators when using block maxima approach.

We are already familiar with the background of extreme value theory, beginning with the limiting distributions of Fisher and Tippett (1928) and advanced theory and applications in Coles (2001). Let (*X_i_*)_*i*≥1_ be i.i.d. random variables with common distribution function *F* ∈ *D*(*G_ξ_*) and corresponding normalisation sequences of constants *a_m_* > 0 and *b_m_* such that:

$$\underset{m\to \infty }{\lim}F^{m}(a_{m}x+b_{m})=F_{\xi }(x),x\in ℜ,$$ [Eqn 1]

where *ξ* is the extreme value index. Let *M_k,m_* = max (*X*_(*k*−1)*m*+1_,…, *X_km_*), *k* ≥1, hence, *n = m × k* observations are divided into *k* blocks of size *m,* where *n* is the total number of observations (Ferreira & De Haan 2015). Then there exists a non-degenerate function *G* such that for a fixed block length *m* ≥ 1, the variables (*M_k,m_*)_*k*≥*1*_ are i.i.d. with distribution function *F^m^* such that:

$$\begin{array}{l} P\left(\frac{M_{k,m}-a_{m}}{b_{m}}\right)=\underset{m\to \infty }{\lim}F^{m}(a_{m}x+b_{m})\\ \to G_{\xi }(x),\text{as}n,k,m\to +\infty \end{array}$$ [Eqn 2]

As suggested by Eqn (2), an approximation of the distribution of *M_k,m_,* or the distribution function *F^m^*, is the GEV distribution with parameters *a_m_, b_m_* and *ξ* estimated by the ML method in the study (Dombry 2013; Ferreira & De Haan 2015). The GEV cumulative distribution function, *G,* is given in Eqn (3) as:

$$G(\mu ,\sigma ,\xi ;x)=\left\{ \begin{array}{l} \exp\left(-{\left(1+\xi \frac{x-\mu }{\sigma }\right)}^{-1/\xi }\right),1+\xi \frac{x-\mu }{\sigma }>0,\xi \neq 0,\\ \exp\left(-\exp\left(-\frac{x-\mu }{\sigma }\right)\right),x\in ℜ,\xi =0 \end{array}\right.$$ [Eqn 3]

where *µ, σ* and *ξ* are the location, scale and shape parameters, respectively. The model in Eqn (3) is the time-homogeneous GEV model. We shall call it model *M*_0_ and it shall be used as the reference model such that all other extended models are compared to it for their significance.

The log-likelihood function for the GEV in Eqn (3) for the case *ξ* ≠ 0 is given by Eqn (4):

$$\begin{array}{l} l(\mu ,\sigma ,\xi ;x)=-klog\sigma -(1/\xi +1)\\ \sum_{i=1}^{k}\log{\left[1+\xi \left(\frac{x-\mu }{\sigma }\right)\right]}_{+}-\sum_{i=1}^{k}{\left[1+\xi \left(\frac{x-\mu }{\sigma }\right)\right]}_{+}^{-1/\xi } \end{array}$$ [Eqn 4]

where *k* is the number of blocks (years) and *x* is AM.

Now consider the time-dependent GEV model, call it *M*_1_, with a linear trend in the location and the scale parameter such that *µ*(*t*) = *µ*_0_ + *µ*_1_ *t*, log *σ*(*t*) = *σ*_0_+*σ*_1_ *t*, and *ξ*(*t*) = *ξ* where *t* is time in years, then the general model is given in Eqn (5):

$$G\left(\mu (t),\sigma (t),\xi (t);x,t\right)=\left\{ \begin{array}{l} \exp\left(-{\left(1+\xi \frac{x-(\mu_{0}+\mu_{1}t)}{\exp(\sigma_{0}+\sigma_{1}t)}\right)}^{-1/\xi }\right),\\ 1+\xi \frac{x-\mu (t)}{\exp(\sigma_{0}t+\sigma_{1}t)}>0,\xi \neq 0,\\ \exp\left(-\exp\left(-\frac{x-(\mu_{0}+\mu_{1}t)}{\exp(\sigma_{0}+\sigma_{1}t)}\right)\right),\\ x\in ℜ,\xi =0 \end{array}\right.$$ [Eqn 5]

The log-likelihood function of model *M*_1_ for the case *ξ* ≠ 0 is given in Eqn (6) as:

$$\begin{array}{l} l(\mu_{0},\mu_{1},\sigma_{0},\sigma_{1},\xi ;x,t)=-klog\sigma -(1/\xi +1)\\ \sum_{i=1}^{k}\log{\left[1+\xi \frac{x-(\mu_{0}+\mu_{1}t)}{\exp(\sigma_{0}+\sigma_{1}t)}\right]}_{+}\\ -\sum_{i=1}^{k}{\left[1+\xi \frac{x-(\mu_{0}+\mu_{1}t)}{\exp(\sigma_{0}+\sigma_{1}t)}\right]}_{+}^{-1/\xi } \end{array}$$ [Eqn 6]

with the usual replacement when *ξ* = 0. The R package *ismev* is used to estimate the parameters of the GEV models (Heffernan & Stephenson 2015; R Core Team 2013).

In the present study, we also propose three more models, *M*_2_, *M*_3_, and *M*_4_. Model *M*_2_ has a linear trend in the location parameter such that *µ*(*t*) = *µ*_0_+ *µ*_1_(*t*), *σ*(*t*) = *σ* and *ξ*(*t*) *= ξ,* and hence model *M*_2_ and its log-likelihood are of the form *G*(*µ*(*t*), *σ, ξ*; *x, t*) and *l*(*µ*_0_, *µ*_1_, *σ, ξ; x, t*). As for the other two models, *M*_3_ has a linear trend in the scale parameter and *M*_4_ has a nonlinear quadratic trend in the location parameter such that *µ*(*t*) = *µ*, log *σ*(*t*) = *σ*_0_ + *σ*_1_*t, ξ*(*t*) = *ξ* and *µ*(*t*) = *µ*_0_ + *µ*_1_*t* + *µ*_2_*t*^2^, *σ*(*t*) = *σ, ξ*(*t*) = *ξ* for models *M*_3_ and *M*_4_, respectively. The model for *M*_3_ and its log-likelihood are of the form *G*(*µ, σ*(*t*), *ξ*; *x, t* and *l*(*µ, σ*_0_, *σ*_1_, *ξ*; *x, t*), respectively, whilst the model for *M*_4_ and its log-likelihood are of the form *G*(*µ*(*t*), *σ, ξ*; *x, t*) and *l*(*µ*_0_, *µ*_1_, *µ*_2_, *σ, ξ*; *x, t*), respectively.

### Model choice

One important question to answer is whether the non-stationary model provides an improvement in fit over the time-homogeneous model *M*_0_; that is, is it worthwhile to have the non-stationary model? The ML estimation of nested models uses a simple procedure called the deviance (*D*) statistic to compare one model against the other. In the study, the time-homogeneous GEV model, *M*_0_, is a special case of the time-dependent models *M*_1_, *M*_2_, *M*_3_ and *M*_4_. In general, consider *M*_0_ ⊂ *M*_i,∀i=1,2,3,4_, then we define deviance statistic, *D*, as in Eqn (7):

$$D=2\left\{l_{i}(M_{i})-l_{0}(M_{0})\right\},$$ [Eqn 7]

where *l_i_*(*M_i_*) and *l*_0_(*M*_0_) are the maximised negative log-likelihood for models *M*_*i*,∀*i*=1,2,3,4_ and *M*_0_, respectively. *D* has a chi-square (χ^2^_*k*,α_) asymptotic distribution, with *k* degrees of freedom tested at *α* (=0.05 or 5%) level of significance, where *k* is the difference in dimensionality (or difference in number of parameters) of *Mi* and *M*_0_. Thus, *D* is compared to critical values of *χ*^2^_*k*,*α*_, where *D* > χ^2^_*k*,*α*_ suggests that model *M_i_* explains substantially more of the variability in the data than model *M*_0_.

## Results and discussion

In order to avoid presenting too many tables in the article, only tables for the AM1 time series data are presented for each of the three sites Chokwe, Combomune and Sicacate in Tables 1, 2 and 3 respectively. The results for the AMS moving sums for each site are only discussed in detail if there is discrepancy with the AM1 results, else they are simply mentioned if there is consistency. The interested reader can obtain results for the AMS moving sums upon request from the corresponding author. The order of the models is maintained for the AMS moving sums, for example, for AM2 time series data model *M*_1_ still refers to a time-dependent GEV model with a linear trend in both the location and scale parameters as in AM1.

**TABLE 1:** Annual daily maximum time-dependent generalised extreme value models for Chokwe for the period 1951–2010.

Model	${\hat{\mu }}_{0}$	${\hat{\mu }}_{1}$	${\hat{\mu }}_{2}$	${\hat{\sigma }}_{0}$	${\hat{\sigma }}_{1}$	$\hat{\xi }$	Maximised negative log-likelihood
*M*_0_	4.248	0	0	1.785	0	−0.081	126.313
*M*_1_	4.222	−0.0003	0	2.204	−0.015	−0.041	125.802
*M*_2_	4.294	−0.0015	0	1.787	0	−0.081	126.308
*M*_3_	4.212	0	0	2.205	−0.015	−0.040	125.802
*M*_4_	4.237	−0.0002	0.0000	1.784	0	−0.080	126.310

**TABLE 2:** Annual daily maximum time-dependent generalised extreme value models for Combomune for the period 1966–2010.

Model	${\hat{\mu }}_{0}$	${\hat{\mu }}_{1}$	${\hat{\mu }}_{2}$	${\hat{\sigma }}_{0}$	${\hat{\sigma }}_{1}$	$\hat{\xi }$	Maximised negative log-likelihood
*M*_0_	5.163	0	0	1.660	0	−0.124	90.740
*M*_1_	5.394	−0.012	0	2.321	−0.033	−0.045	88.060
*M*_2_	5.445	−0.011	0	1.685	0	−0.150	90.614
*M*_3_	5.034	0	0	2.268	−0.031	−0.043	88.276
*M*_4_	5.338	−0.000	−0.000	1.681	0	−0.146	90.627

**TABLE 3:** Annual daily maximum time-dependent generalised extreme value models for Sicacate for the period 1952–2010.

Model	${\hat{\mu }}_{0}$	${\hat{\mu }}_{1}$	${\hat{\mu }}_{2}$	${\hat{\sigma }}_{0}$	${\hat{\sigma }}_{1}$	$\hat{\xi }$	Maximised negative log-likelihood
*M*_0_	6.151	0	0	3.328	0	−0.454	148.547
*M*_1_	6.901	−0.012	0	1.813	0.061	−0.682	144.306
*M*_2_	5.499	0.025	0	3.443	0	−0.526	148.279
*M*_3_	6.675	0	0	1.966	0.055	−0.693	144.413
*M*_4_	5.887	0.000	0.0003	3.396	0	−0.499	148.373

### Chokwe models

Consider the pair of models (*M*_0_, *M*_1_) from Table 1, where *M*_0_ is taken as the reference model, χ^2^_2,0.05_ = 5.991, *D* = 2(−125.802−(−126.313)) = 2(126.313−125.802) = 1.022 and the likelihood ratio test for *m*_1_= 0 has *p*-value = 0.4928 and for σ_1_= 0 has *p*-value = 0.1676 Because *D* is too small compared to the critical value (5.991) and the likelihood ratio test is not significant at the 5% level of significance (*p*-value > 0.05) for both the location and scale parameters, it clearly shows that the non-stationary model is not important and does not give any improvement in fit over the time-homogeneous GEV model. Similar insignificant results were obtained for the AMS moving sums AM2, AM5, AM7, AM10 and AM30 for model *M*_1_.

The other pairs from Table 1, (*M*_0_, *M*_2_) and (*M*_0_, *M*_3_), have *D* = 0.01 and 1.022, respectively, with a critical value of *χ*^2^_1,0.05_ = 3.841 for both pairs. The likelihood ratio test for *µ*_1_ = 0 has *p*-value =0.4591 and *σ*_1_ = 0 has *p*-value = 0.1653 for *M*_2_ and *M*_3_, respectively, which is insignificant for both models at the 5% level of significance. The *D* statistic is again too small (< 3.841) for both models, implying that both models do not provide any improvement in fit over the time-homogeneous GEV model. Similar insignificant results were exhibited for the AMS moving sums AM2, AM5, AM7, AM10 and AM30 for models *M*_2_ and *M*_3_.

The quadratic model pair (*M*_0_, *M*_4_) in Table 1 has a *D* statistic value of 0.006 with a critical value of *χ*^2^_2_,_0.05_ = 5.991, implying that model *M*_4_ does not provide any improvement in fit to justify its importance over the time-homogeneous model. The likelihood ratio tests for *µ*_1_ = 0 and *µ*_2_ = 0 are also not significant at the 5% significance level (*p*-value > 0.05). Again, similar results were obtained for AM2, AM5, AM7, AM10 and AM30.

**FIGURE 2:**
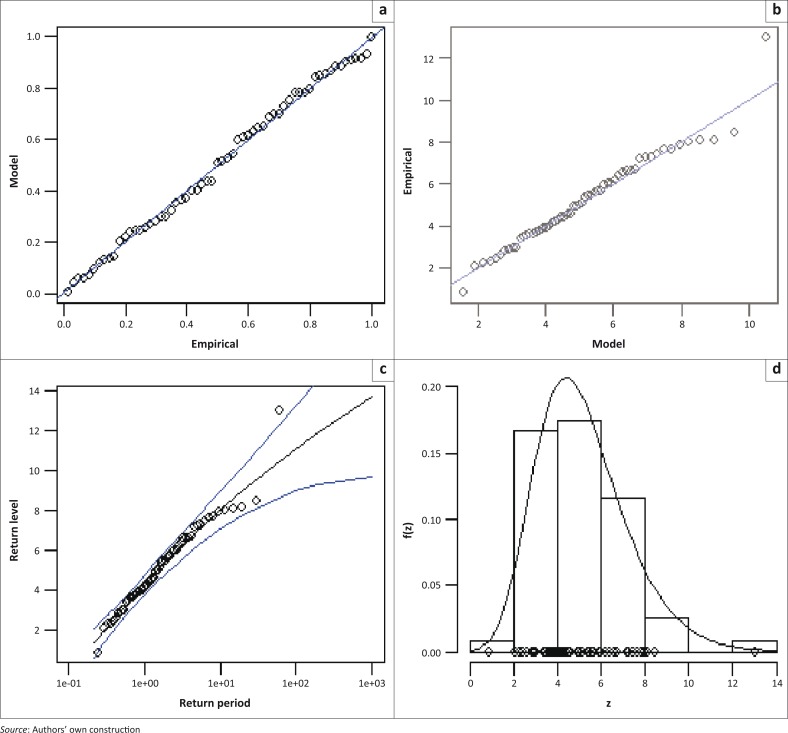
Diagnostic plots for the time-homogeneous generalised extreme value model at Chokwe hydrometric station: (a) Probability plot; (b) Quantile plot; (c) Return level plot; (d) Density plot.

In general, the results from Chokwe showed that the prevailing model for the site is the time-homogeneous GEV model given by Eqn (3). In other words, time is not an important factor for the AMS data at Chokwe. The general model estimate for Chokwe is given in Eqn (8):

$$\begin{array}{l} G(\mu ,\sigma ,\xi ;x)=exp\left(-{\left(1+\frac{-0.081\left(x_{i}-4.248\right)}{1.785}\right)}^{1/0.081}\right),\\ 1+\frac{-0.081(x_{i}-4.248)}{1.785}>0 \end{array}$$ [Eqn 8]

where *x* = *x*_*i*,∀*i*=1,2,…,*k*_ is the AM flood height. The diagnostic plots for the time-homogeneous model in Eqn (8) are presented in Figure 2. The diagnostic plots in Figure 2 show that the model is of good fit, with the exception of the year 2000 flood height which falls slightly outside the confidence limits.

### Combomune models

We start by considering the pair (*M*_0_, *M*_1_) from Table 2 with *χ*^2^_2_,_0.05_ = 5.991, *D* = 5.36, and likelihood ratio test for *µ*_1_ = 0 has *p*-value = 0.2570. and for *σ*_1_= 0 has *p*-value = 0.0117. These results show that the linear trend in location parameter is not significant at the 5% significance level (*p*-value > 0.05), whilst the linear trend in scale parameter is significant (*p*-value < 0.05) in the model. In other words, the scale parameter is time dependent whilst the location parameter is time-homogeneous. However, the *D* statistic (5.36) is less than the critical value of 5.991 at 2 degrees of freedom, which implies that the non-stationary model *M*_1_ is not worthwhile compared to the time-homogeneous GEV model in Eqn (3). The same conclusions were reached for the moving sums of AM2, AM5, AM7, AM10 and AM30.

We now consider the pairs (*M*_0_, *M*_2_) and (*M*_0_, *M*_2_) from Table 2. The critical value for both pairs is *χ*^2^_1,0.05_ = 3.841 with respective *D* statistic values of 0.252 and 4.928 for the two pairs. The likelihood ratio test for *µ*_1_ = 0 has *p*-value = 0.3092 and *σ*_1_ = 0 has *p*-value = 0.0141 for models *M*_2_ and *M*_3_, respectively. These results show that model *M*_2_ is not significant at the 5% significance level (*p*-value > 0.05) and is not worthwhile because *D* statistic value (0.252) is too small compared to the critical value (3.841). On the other hand, model *M*_3_ is significant at the 5% significance level (*p*-value < 0.05) and provides an improvement in fit over the time-homogeneous GEV model because the *D* statistic value of 4.928 (> 3.841) is significantly large. Similar findings were obtained for all the AMS moving sums.

The nonlinear quadratic model pair (*M*_0_, *M*_4_) has a *D* statistic of 0.226, which is too small compared to the critical value of 5.991 with two degrees of freedom. The likelihood ratio tests for *µ*_1_ = 0 and *µ*_2_= 0 are not significant at the 5% significance level (*p*-value > 0.05). Thus, the nonlinear quadratic model *M*_4_ is neither significant nor worthwhile over the time-homogeneous GEV model. Likewise, the same conclusions were reached for all the AMS moving sums.

**FIGURE 3:**
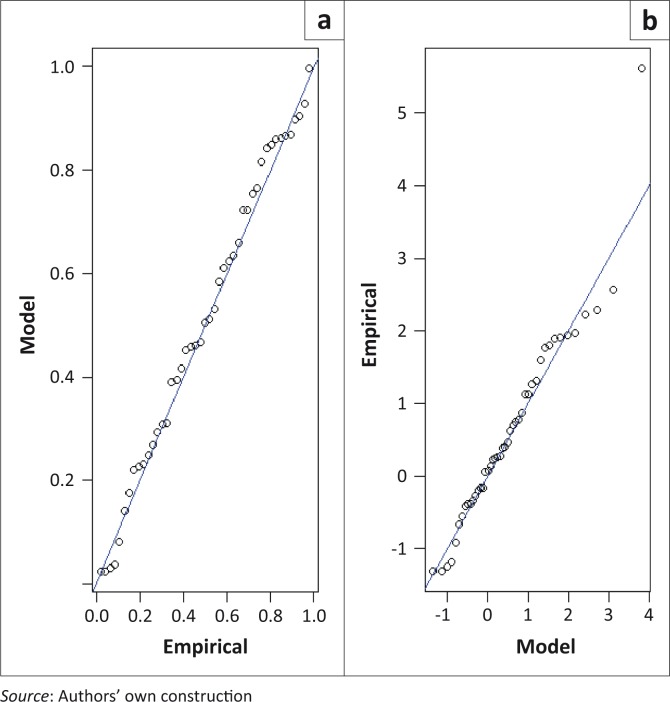
Diagnostic plots for the time-heterogeneous generalised extreme value model with a trend in the scale parameter at Combomune hydrometric station: (a) Residual probability plot; (b) Residual quantile plot.

Overall, the final model for Combomune is the non-stationary model, *M*_3_, with a linear trend in the scale parameter of the GEV. The general model for Combomune is given in Eqn (9):

$$\begin{array}{l} G(\mu ,\sigma (t),\xi ;x,t)=exp\left(-{\left(1+\frac{-0.043\left(x_{i}-5.034\right)}{\exp(2.268-0.031t_{i})}\right)}^{1/0.043}\right),\\ 1+\frac{-0.043(x_{i}-5.034)}{\exp(2.268-0.031t_{i})}>0 \end{array}$$ [Eqn 9]

where *t_i_* = *τ_i_*−1965, *τ_i_ =* 1966, 1967, … and *t_i_* = 1, 2, 3, … is time in years and *x* = *x*_*i*,∀*i*=1,2,…,_ is the AM flood height. The diagnostic plots for the time-heterogeneous model in Eqn (9) are presented in Figure 3. The residual probability plot suggests a good fit to the data.

### Sicacate models

The model pair (*M*_0_, *M*_1_) from Table 3 has *χ*^2^_2,0.05_ = 5.991 and a *D* statistic value of 8.482. The likelihood ratio test for *µ*_1_ = 0 has *p*-value = 0.3217 and *σ*_1_ = 0 has *p*-value = 0.0045, which indicates that the linear trend in location parameter is not significant at the 5% significance level (*p*-value > 0.05), whereas the linear trend in scale parameter is highly significant (*p*-value < 0.05) in the model. Because the *D* statistic value (8.482) is greater than the critical value of 5.991, we conclude that model *M*_1_ provides an improvement in fit over the time-homogeneous GEV model; that is model *M*_1_ is worthwhile. These findings are consistent with findings from AMS moving sums AM2, AM5, AM7, AM10 and AM30.

The model pairs (*M*_0_, *M*_2_) and (*M*_0_, *M*_3_) in Table 3 share a critical value of *χ*^2^_1,0.05_ = 3.841 with a *D* statistic value of 0.536 and 8.268 for *M*_2_ and *M*_3_ respectively. The likelihood ratio test for *µ*_0_ = 0 has *p*-value = 0.2638 and *σ*_1_= 0 has *p*-value = 0.0013 This indicates that model *M*_2_is insignificant at the 5% level of significance (*p*-value > 0.05) and not worthwhile (*D* < 3.841), whilst model *M*_3_ is highly significant at the 5% significance level (*p*-value < 0.05) and provides an improvement in fit over the time-homogeneous GEV model, with a large *D* statistic value of 8.268 (> 3.841). These findings are also consistent with findings from all the AMS moving sums.

The nonlinear quadratic model pair (*M*_0_, *M*_4_) in Table 3 has a *D* statistic value of 0.348 with a critical value of *χ*^2^_2,0.05_ = 5.991 The likelihood ratio test for *µ*_1_ = 0 has *p*-value = 0.4991 and *µ*_2_ = 0 has *p*-value < 0.0001 This implies that the linear trend term in the location parameter is not significant at the 5% level of significance (*p*-value > 0.05) whilst the quadratic trend term in location parameter is highly significant (*p*-value < 0.0001). However, the overall nonlinear quadratic model is not worthwhile because the *D* statistic value of 0.348 is too small compared with the critical value of 5.991. Again, these findings are consistent with findings from all the AMS moving sums.

**FIGURE 4:**
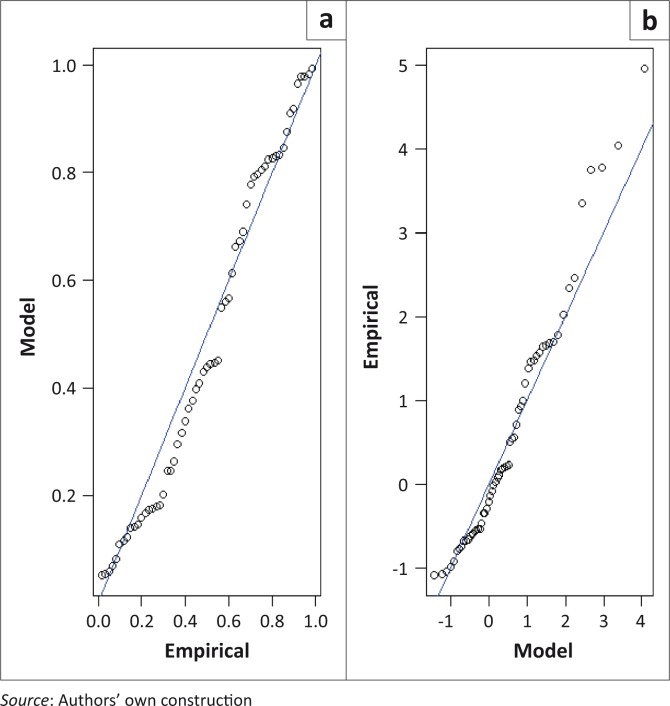
Diagnostic plots for the time-heterogeneous generalised extreme value model with a trend in the scale parameter at Sicacate hydrometric station: (a) Residual probability plot; (b) Residual quantile plot.

**FIGURE 5:**
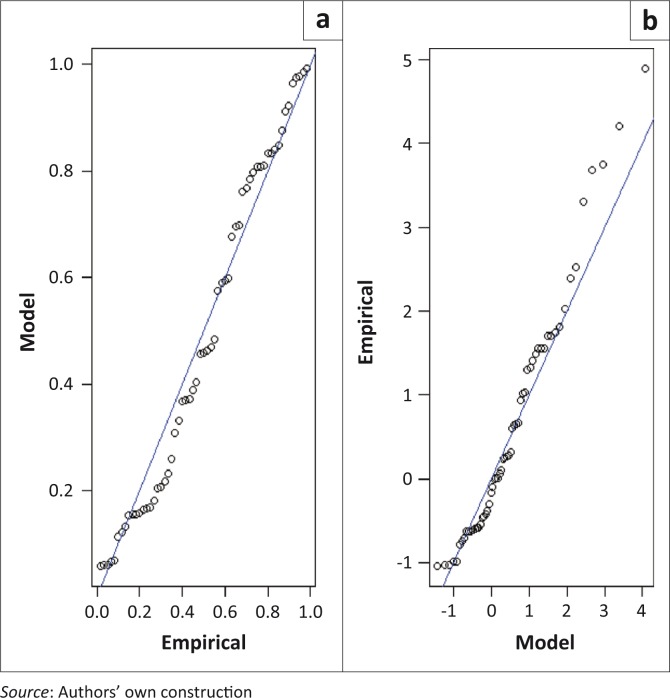
Diagnostic plots for the time-heterogeneous generalised extreme value model with a trend in both the location and scale parameters at Sicacate hydrometric station: (a) Residual probability plot; (b) Residual quantile plot.

We now have two competing ‘good’, non-stationary, linear, time-dependent models for Sicacate. To identify the most appropriate of these models, we rate the one with the smaller standard errors and the smaller *p*-value as the most appropriate model. In this case, model *M*_3_ has a smaller *p*-value in the slope of the scale parameter 0.0013 compared to 0.0045 for *M*_1_, and the standard errors for *M*_3_ are much smaller than those of *M*_1_ for example 0.47428 compared to 0.64696 for *µ*ˆ_0_ and 0.01722 compared to 0.02249 for scale slope *σ*ˆ_1_ for *m*_3_ and *M*_1_, respectively. Therefore, the non-stationary linear trend in scale GEV model for Sicacate is given in Eqn (10) and the alternative model is given in Eqn (11) as follows:

$$\begin{array}{l} G(\mu ,\sigma (t),\xi ;x,t)=exp\left(-{\left(1+\frac{-0.693\left(x_{i}-6.675\right)}{\exp(1.966+0.055t_{i})}\right)}^{1/0.693}\right),\\ 1+\frac{-0.693(x_{i}-6.675)}{\exp(1.9660+0.055t_{i})}>0 \end{array}$$ [Eqn 10]

where *t_i_* = *τ_i_* –1951, *τ_i_* = 1952, 1953,… and *t_i_* = 1, 2, 3,… is time in years and *x* = *x*_*i*,∀*i*=1,2,…,k_ is the AM flood height. The alternative non-stationary linear trend in location and scale GEV model is given as follows:

$$\begin{array}{l} G(\mu (t),\sigma (t),\xi ;x,t)\\ =exp\left(-{\left(1+\frac{-0.682\left(x_{i}-(6.901-0.012t_{i})\right)}{\exp(1.813+0.061t_{i})}\right)}^{1/0.682}\right) \end{array}$$ [Eqn 11]

where *t_i_* = *τ_i_* – 1951, *τ_i_ =*1952,1953,… and *t_i_* = 1,2,3,… is time in years and *x* = *x*_*i*,∀*i*=1,2,…,*k*_ is the AM flood height. The diagnostic plots for the time-heterogeneous models in Eqn (10) and Eqn (11) are presented in Figure 4 and Figure 5, respectively. The residual probability plots for both models suggest a good fit to the data.

The interesting findings are that whilst most studies in other regions have found a dominant linear trend in the location parameter of the GEV distribution for some rivers (e.g. Katz *et al*. 2002), the study has found no evidence of a significant linear trend in the location parameter of the GEV distribution for the LLRB of Mozambique. On the other hand, the study has revealed a dominant time-dependent scale parameter for the river at Combomune upstream and Sicacate further downstream. The study also revealed evidence of a highly significant nonlinear trend, quadratic term, in the location parameter at Sicacate, although the complexity of the overall model was not worthwhile with reference to the time-homogeneous GEV model. The findings in the study are in full support of a previous study by Aich *et al*. (2014), who used a geoscientific model called eco-hydrological SWIM model to compare the climate change impacts on streamflow in four large African basins including the Niger, upper Blue Nile, Oubangui and Limpopo and found the Limpopo basin to be highly sensitive to climate change variability. The results obtained in the study complemented by those of Aich *et al*. (2014) explain the reason for the increased frequency of extreme floods in the LLRB of Mozambique, which can be attributed to the variability in climatic conditions. The time-dependent GEV models developed in the study are worth considering by the Mozambican government and its partners, as well as its neighbours, in their policies and decision making. Chokwe Irrigation Scheme, the largest irrigation scheme in Mozambique is situated in the LLRB, making the basin the backbone of the country’s economy, which is mainly characterised by agriculture and fishing.

## Conclusion

The study considered the use of statistics of extremes in a changing climate for the LLRB of Mozambique. Three hydrometric stations representing three sites along the lower Limpopo River were considered for the study. The ML estimation method was used to estimate the parameters of the GEV distribution in the presence of a trend covariate. The study has revealed the importance of considering non-stationary linear and nonlinear trend models when using statistics of extremes in a changing climate as these models provide an improvement in fit over the time-homogeneous models. This improvement in fit is very important for the planning and policy-making of the government of Mozambique and its partners in the LLRB, where the largest irrigation scheme of the country is situated. The importance of the developed models is attributed to the fact that these non-stationary models take into account the reasons for increased frequency of floods in the basin. Once the government and its partners are fully aware of the reasons behind the increased frequency of floods in the basin, their planning can be much improved.

The study has successfully identified the prevailing models at the three sites such that Chokwe is the only site with a time-homogeneous GEV model. This can be attributed to the fact that some of the water at the site is diverted to the Chokwe Irrigation Scheme for irrigation purposes. The other two sites Combomune and Sicacate have a prevailing non-stationary GEV model with a dominant linear trend in the scale parameter. The site of Sicacate has an alternative non-stationary model with a linear trend in both the location and scale parameters of a GEV distribution. The prevailing models established in the study are consistent with cumulative (or moving sums) AMS flood flows and therefore appear reliable to use for flood frequency analysis in the basin. The use of the identified time-dependent GEV models with a trend in the scale parameter in the basin would also reduce the sensitivity of the frequency of floods, which is known to vary with changes in the scale parameter and therefore lead to more reliable estimates in the frequency of floods.

Future studies will attempt to advance the study to consider non-stationary generalised Pareto distributions, Bayesian inference and Markov chain Monte Carlo methods in a changing climate for the lower Limpopo River of Mozambique. Covariates in the form of cycles and/or a physical variable such as a dummy variable indicating the occurrence of cyclones in the region will also be considered in future studies involving statistics of extremes in a changing climate.
